# Wild-captive interactions and economics drive dynamics of Asian elephants in Laos

**DOI:** 10.1038/s41598-017-13907-x

**Published:** 2017-11-01

**Authors:** Gilles Maurer, Benjamin S. Rashford, Vatsana Chanthavong, Baptiste Mulot, Olivier Gimenez

**Affiliations:** 1 Centre d’Ecologie Fonctionnelle et Evolutive (UMR 5175), CNRS – Université de Montpellier – Université Paul-Valéry Montpellier – EPHE, campus CNRS, Montpellier, France; 2 Zooparc de Beauval & Beauval Nature, Saint-Aignan, France; 3 Department of Agricultural and Applied Economics, University of Wyoming, Laramie, USA; 4 Department of Livestock and Fisheries, Ministry of Agriculture and Forestry, Vientiane, Lao PDR; 5 ElefantAsia non-profit organization, Paris, France

## Abstract

The interactions between wild and captive populations of Asian elephants (*Elephas maximus*) persist in most countries of the species distribution, notably through the reproduction between captive females and wild males. However, these complex interactions have been poorly studied, despite their relevance for conservation of this endangered species. Laos has a centuries-long tradition of raising Asian elephants. Besides being cultural icons, captive elephants are inextricably linked to economics through their work in forestry. Using an ecological-economic model, we investigated the effect of socio-economic strategies on fecundity of the Lao population whose dynamics is shaped by human practices. We demonstrated that fecundity is impacted by: i) the dynamics of the wild elephant pool through mating of captive females by wild males, and ii) the financial incentive of elephant owners to breed their animals. As a result, we expect fecundity to rise in response to increases in elephant prices. The captive population will tend towards an asymptotic limit determined by the wild pool growth rate. However, the population will tend to extinction if exports continue. Our ecological-economic approach, by accounting for economic incentives, allows us to predict new equilibria that can serve as a baseline for designing sustainable management strategies for the species.

## Introduction

The population of Asian elephants, *Elephas maximus*, is in overall decline and classified as endangered by the International Union for Conservation of Nature. Total population abundance ranges from 30,000 to 50,000 individuals; however, these figures are probably overestimates^1–3^. One-quarter to one-third of the population consists of captive elephants^4,5^. Captive elephants were traditionally sourced from wild populations though bans on wild capture have been in place since the 1970s^6–8^. These so-called “domestic” elephants are, however, not a domesticated species and are similar to their wild counterparts in terms of genetics and behaviour^9^.

The interactions between wild and captive populations persist in most countries of the species distribution, mainly through reproduction between captive females and wild males. In Laos, India and Myanmar, captive females are released in the forest and pair with wild males^9–12^. Nearly 80% of calves born in captivity in Laos during the past decade are from wild genitors from the Nam Pouy Protected Area (V. Chanthavong, pers. comm.). In Myanmar, live capture of wild elephants has been used to sustain the captive pool. This practice may, however, pose a serious threat to the survival of the country’s wild population^4^. Elephant calves also have great appeal to tourists and, hence, are highly valued^13^. With corruption, a lack of law enforcement, and deficient registration systems of captive individuals in many Asian countries, the incentive is high to illegally capture and smuggle calves to supply the tourism sector^14^. As a result, the captive population partially relies on wild males for breeding, while the wild pool is threatened by illegal captures to supplement the captive pool. Both captive and wild pools also share common threats and conservation challenges, such as habitat destruction, poaching, increased conflicts with local human populations, and risks of inbreeding depression^12^. Despite their relevance for the conservation of the species, the complex interactions between wild and captive pools have been poorly studied.

The understanding of Asian elephant demography and ecology has recently improved thanks to a longitudinal study of a large semi-captive population in Myanmar. For example, it has been shown how life histories could impact reproductive success, survival or senescence^15–18^. Climate variations also impact age-specific survival of Asian elephants^19,20^. Besides survival, large variations in average fecundity rates have been observed in different populations across the region and over time, with a fecundity rate of 0.023 (2.3% of breeding females) in Laos^21^, 0.083 in Myanmar^11^, and varying from 0.095 to 0.155 for a captive elephant population in India over the past century^10^. No systematic assessment explains why the captive birth rates are highly variable and lower than birth rates observed in wild populations. It has been hypothesized to be the consequence of management and husbandry practices, which in effect place breeding secondary to work^4^.

The alteration of vital rates in the dynamics of wild-captive Asian elephant populations illustrates the intricacies of mixing natural and human-induced mechanisms^22^. Our objective was to assess the effect of these interactions on the viability of Asian elephant populations using an ecological-economic modelling approach with Lao populations as a case study.

Lao captive elephants are traditionally used as draught animals in the logging industry and share the same habitat as their wild conspecifics. In the last twenty years, the intensification of elephants’ workload has affected the fecundity of the population, leading to a sharp decline from 1,000 to 540 individuals^21^. The long gestation and lactation period for female elephants is not compatible with work^4^. Owners consider breeding to represent a loss of income for nearly three years and therefore avoid breeding their elephants, preferring the high incomes generated by the logging industry^4^. Meanwhile, the contiguous Nam Pouy wild population has experienced a sharp decrease from 400–500 individuals in 1990 to 60–80 in 2010^23–25^, further hindering the reproductive potential between wild males and captive females. Finally, the Lao captive population has also been affected by the withdrawal of individuals through the export of captive juveniles to third countries. These exports violate the Convention on International Trade in Endangered Species of Wild Fauna and Flora (CITES) despite its ratification by the Lao government in 2014. While exports are highly regulated, the trade of captive elephants is unregulated on the domestic market^26^.

To develop a demographic model incorporating economic constraints, we considered the processes impacting the dynamics of captive elephants with a focus on the determinants of fecundity. Therefore, we developed a model including three components: (i) a demographic component structured by sex and age, including the exports of living captive elephants, to estimate the captive population abundance at a given time; (ii) a wild elephant component to account for the wild male breeding potential; (iii) and an economic component to model the elephant owner’s decision-making process. Finally, we defined several demographic and socioeconomic scenarios to investigate new ecological-economic equilibria that can serve as a basis to design sustainable management strategy for the species.

## Results

### Changes in breeding practices

Breeding practices radically changed since Laos transitioned to an open market economy in 1989. Traditionally, captive elephants were used for occasional village work while spending most of their time in small herds, grazing freely in the village surroundings. Mating of captive females with either captive or wild males was uncontrolled.

In 2015, we conducted 52 semi-structured interviews among elephant owners, keepers, and officials throughout the distribution of captive elephants in Laos – 41 out of 50 respondents indicated difficulties finding a captive mate. Among owners of females, the main reason given for the difficulty in finding a captive male was the absence of captive breeding males (21 out of 43) because “there is no male nearby”, “males are kept far away”, “they (males) are engaged in logging and therefore not available for mating”, or “the few males living nearby” are “too young” or “sterile”. Two other reasons were given equally (11): stud fees being too expensive, and their elephant being too tired for breeding. Owners of males indicated having difficulties finding a captive female for mating because “females are not available” ( 8), “their male is too tired for mating” (8), and “females owners do not want to pay stud fees” (6).

Meanwhile 28 out of 42 respondents considered that mating with a wild male is easier than with a captive one, including all respondents (11) living at the edge of Nam Pouy park and having access to the wild pool. Such a mating system was easier because wild males are “available”, “free of their movements”, “in good health”, “not exhausted unlike the working captive males”, and “do not ask for stud fee payment”.

As a consequence, the reproduction of captive females depends mainly on the presence of wild males in Nam Pouy, and to a lesser extent, on the availability of a captive mate. Unlike in wild populations, the reproduction of captive females is density-dependent towards males.

### How much does an elephant cost?

The captive population dynamics are also driven by the willingness of owners to breed their female. According to economic theory, owners maximize profits, and therefore have to make an economic choice between breeding their female or having them work in the logging industry. The owner estimates how much he can earn by selling a calf after weaning and compares these earnings with the foregone alternative income from logging during the three-year gestation and weaning period.

Our model incorporated the price of an elephant following supply and demand theory – that is, a product (i.e., elephant) is a function of its quantity (i.e., the population). Thus, the price of an elephant can be expressed in terms of the population size given by the demographic component of the model. We associated the sale price given by 26 owners who sold their elephants between 1994 and 2012, to the population size produced by the model at the time of sale. Elephant prices in constant USD were exponentially related to total population (all parameters being different from 0 with p-value < 0.035).

**Figure 1:**
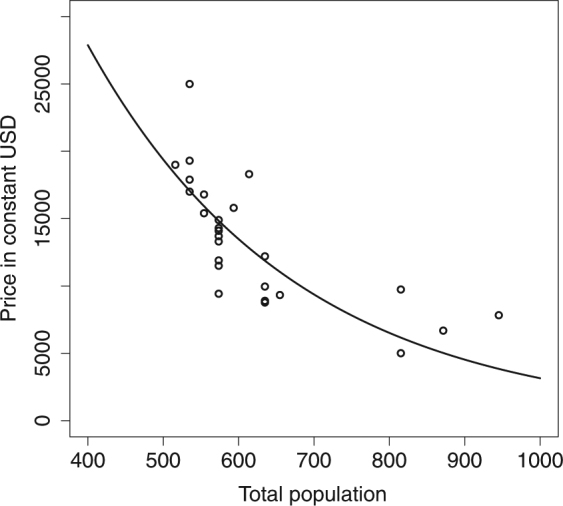
Elephant price is a function of total population abundance Prices are given in constant USD, base year 2000.

The price of an elephant in constant USD increased with the decrease in population (Fig. 1). From 1990 to 2012, the increasing scarcity of elephants was accompanied by a rapid increase in sale prices exceeding 20,000 constant USD^27^. This result illustrates that the relationship between quantity and price is consistent with economic theory. Lower populations (quantity available to the market) lead to higher prices, which lead more owners to conclude it is profitable to breed their elephants rather than using them for logging.

### Population projections depending on ecological-economic scenarios

Using our ecological-economic model, we investigated the impacts of economic and government policies on future trends in the captive population. The government may impact the population dynamics by revising its policy on the controversial export of elephants to third countries and by fighting illegal trade. The government could also reinforce its wild elephant conservation policy by enforcing the law and effectively protecting the Nam Pouy park wild pool. Finally, the government and private stakeholders could promote pro-birth policies for captive elephants (e.g., by compensating owners for foregone logging returns).

Based on the 2012 population census of captive elephants and on estimates of the Nam Pouy wild population, we selected five scenarios that are representative of the different dynamics of the wild pool and levels of exports. All scenarios are based on a price-dependent domestic market. The reference scenario (i) showed the current level of exports of juveniles and a decreasing wild pool at a rate of 8%. The four other scenarios were based on an effective prevention of exports with (ii) a decreasing wild pool, (iii) a stabilized wild pool to investigate the effect of a conservation policy, (iv) a growing wild pool, and (v) a maternity-leave scheme that financially compensates owners for the loss of income during the breeding period. We ran all scenarios for a period of 100 years with stochasticity in the economic growth rate.

**Figure 2:**
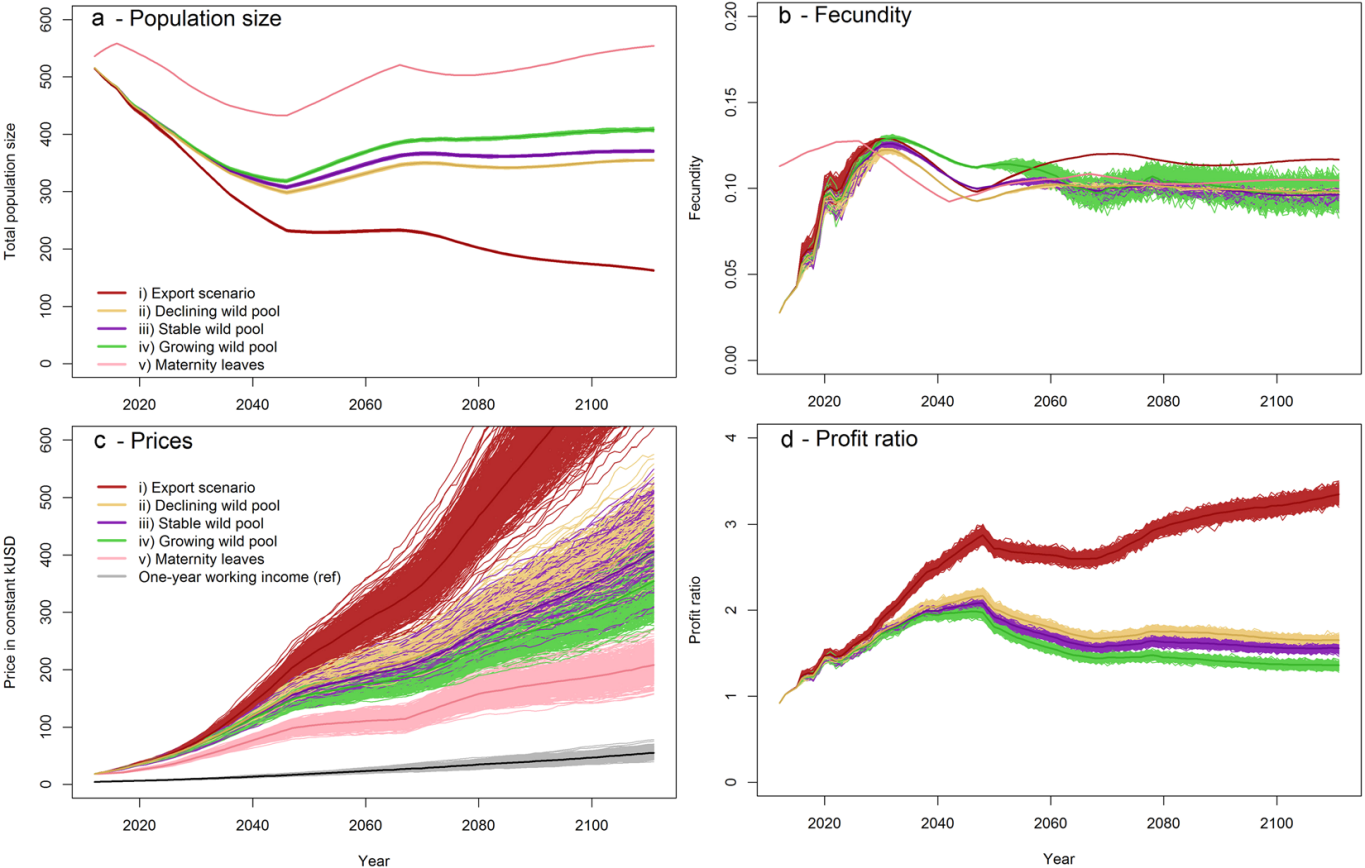
Model projections depending on ecological-economic scenarios (**a**) Population estimates; (**b**) Fecundity; (**c**) Elephant prices in constant thousand USD; (**d**) Profit ratio. The different scenarios within each figure are: (i) Current level of exports and declining wild pool; (ii) No export and declining wild pool at a growth rate of 0.92; (iii) No export and stable wild pool; (iv) No export and growing wild pool at a growth rate of 1.03; (v) Maternity-leave compensation scheme with no export and declining wild pool. The median value of the 500 simulations is shown in contrasted color. The gray line in (d) shows one-year working income as a reference. (**d**) does not show the profit ratio for the maternity-leave scenario (v) as it tends to infinity.

Whatever the scenario, the population decreases during the first 35 years until it recovers from the transient and unstable structure observed in 2012 (Fig. 2a). If current export practices continue (i), the population decline slows, but tends to extinction over the long term with an estimated population of 10 individuals after 480 years. If exports are prevented, the population reaches an asymptotic equilibrium that depends on the dynamics of the wild pool. After 100 years, the captive population reaches an abundance of 355, 371 and 408 individuals for a decreasing (ii), stabilized (iii), or growing (iv) wild pool. Maternity leaves (v) are the only scenario allowing the population to exceed 2012 abundance with 554 individuals. In this case, compensating the owners’ loss of income counteracts the prevailing effect of economic incentives on elephant fecundity.

The population decrease due to exports (i) tends to sharply pull prices upward (Fig. 2c), reaching prices over 800,000 constant USD. The population increases for the other scenarios equalize prices from USD 206,000 [s.d. 20,000] (scenario v) to USD 428,000 [s.d. 39,000] (scenario ii). Consequently, the profit ratio (Fig. 2d) stabilizes respectively at 1.36 [s.d. 0.02] (iv), 1.56 [s.d. 0.02] (iii) and 1.65 [s.d. 0.03] (ii). The rising prices in the export scenario (i) brings the profit ratio to 3.35 [s.d. 0.05] after 100 years. As maternity leaves cover the loss of income, profit ratio of scenario (v) tends to infinity.

Fecundity rates (Fig. 2b) follow the same growing trend as the profit ratio, but are constrained by density dependence in reproduction (see Methods). Export scenario (i) displays the higher fecundity rate of 0.117 followed by the maternity leave (v) fecundity rate 0.105. The fecundity rates in the three other scenarios (ii to iv) are approximately 0.095.

### Changes in demand for elephants

**Figure 3:**
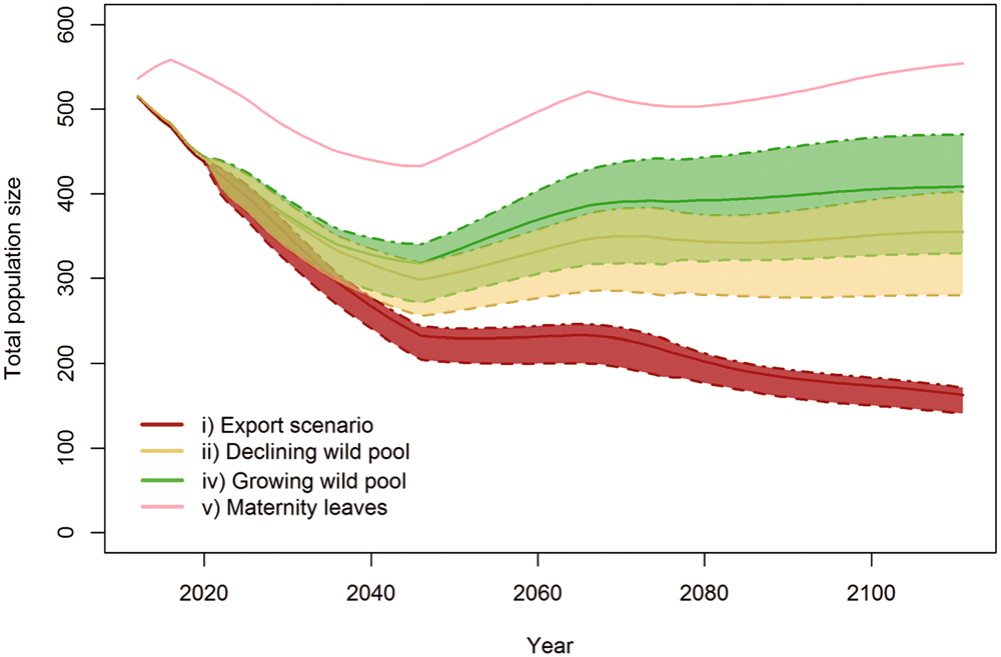
Population size projections under various scenarios of demand variation. (i) Current level of exports and declining wild pool. (ii) No export and declining wild pool at 8% per year. (iv) No export and growing wild pool at 3% per year. (v) Maternity-leave compensation scheme with no export and declining wild pool. Dot-dashed lines give the total population size for demand increase (upper estimate), dashed lines the total population size for demand decrease (lower estimate) and plain lines the total population size with constant demand (as shown in Fig. 2a). Demand variations do not affect maternity-leave scenario (v).

Because we study elephant population dynamics, we built an economic model centered on supply. Variations in supply, the total population, explain price fluctuations given that demand for elephants is proportional to supply. We investigated the effect of a change in demand by applying a 25% decrease and increase in elephant prices. If demand fluctuates over time, using fixed rates allowed us to define a maximum interval bounding the total population estimates under supply and demand equilibrium (Fig. 3).

In the export scenario (i), population estimates range from 141 to 172 for a 25% decrease and a 25% increase in demand for elephants. The declining (ii) and growing (iv) wild pool scenarios have respective population estimates of 280 to 402 and 330 to 470. Once again, population size in the maternity-leave scenario (vi) is not affected by the variation in demand.

### Elasticity analysis

We projected the population out 20 years in each scenario and calculated the elasticity of the resulting population growth rate to model parameters (see Supp. Note 3). The parameter showing the highest elasticity (0.033) is the wild pool growth rate (*grw*) for the growing wild pool scenario (iv). It decreases to 0.021 for the stable wild pool scenario (iii) and 0.005 for the declining wild pool (ii), export (i) and maternity-leaves (v). The female export rate shows a medium-range elasticity (0.012) for the export scenario (i) while it is null for all other scenarios. All other parameters show similar elasticities whatever the scenario with maximum fecundity and sex ratio at a rate of approximately 0.03. Elasticity to female survival rates decline continuously from 0.03 to zero. Elasticity to male survival shows much lower values with a maximum of 0.007 for the 35 years age class. Other model parameters have an elasticity rate lower than 0.001, including all economic parameters.

## Discussion

No study has investigated the effects of socio-economic strategies on the fecundity of an animal population whose dynamics are shaped by human economic practices. We demonstrate that fecundity of captive elephants is impacted by: i) the dynamics of the wild elephant pool through mating of captive females by wild males, and ii) the economic incentives of elephant owners to breed their animals. As a result, we expect fecundity to rise again gradually in response to the rapid increase in the price of elephants. The captive population will tend towards an asymptotic limit determined by the dynamics of the wild pool. In any case, the population will tend to extinction if the government does not stop export practices at current rates. Elephant exports, mostly to Chinese zoos and tourism enterprises, threaten the survival of the Laotian population.

Our model illustrates the strong interactions between the wild and captive pools. The value of captive elephants for conservation have been recognized, not only because of their numbers and the threat that their capture could pose to wild pools, but also for their potential roles in reintroduction, patrolling, human-elephant conflict mitigation, and scientific research^9,28^. Beyond the risks of extinction of wild populations, we showed that wild pool dynamics directly impact the survival of the captive population through crossbreeding. The elasticity analysis confirms the influential contribution of a stable or growing wild pool to the growth of the captive population. The Nam Pouy wild pool was modelled using a geometric growth rate, based on published data^23–25^; however, these data are scarce and approximate. A better estimate and monitoring of the abundance of this population would reinforce the recognition of how dynamics of the wild pool affect the demography of the captive pool. For the time being, it seems urgent to strictly protect the wild populations and consider organizing new wild-captive breeding areas in Laos. Policy-makers should also consider the side-benefits of a strict conservation strategy for wild elephants in regards to the cultural and economic role played by captive elephants.

Our ecological-economic approach also emphasizes the impacts of human practices on the dynamics of the captive elephant pool. Several models have been developed to search for potential balance between ecological and economic dynamics. Most of these models favored a macroeconomic approach – searching for a global equilibrium between total profit maximization and population survival^29–31^. In our case, we directly linked fecundity with owner profit maximization. In the absence of reliable macroeconomic data, this microeconomic approach allowed us to model the owner’s decision-making process and study its impact on species’ demography. However, our elephant price dataset was derived from data for a population between 530 and 900 individuals. Therefore, the estimated price of elephants in a population of less than 200 individuals remains questionable, being far from the original data range. This was one reason we limited our projections to 100 years.

As shown by our model, changes in handling practices have long-term effects on population structure and dynamics. In 2012, the population was already transient and unstable, with a strong bias in favour of adults over 20 years old (see the age pyramid in Methods – Model calibration). As a result, the model indicates it will take 35 years before the population grows again. Long-lived species, such as the Asian elephant, are highly sensitive to population inertia^32^, with long periods before the population may recover from any change in a vital rate. Further investigation of such transient status and its consequences on elephant population dynamics are needed. But our model demonstrates that the commodification of captive elephants and its accompanying changes in handling practices have probably dominated the ecological dynamics of this population, as shown by the short-term elasticity analysis (Supp. Note 3).

Our maternity-leave compensation scheme provides additional insights into the conservation questions surrounding captive and semi-captive populations. If owners are compensated for their loss of income during breeding, the steep economic trade-off between using an elephant for breeding versus work is removed, the elasticity of all economic parameters being null under this scenario. By covering the opportunity cost of breeding, the maternity-leave scheme we analysed allows the population to exceed its 2012 abundance. Such a policy could be funded either by a tax on elephant incomes or directly by the tourism industry, for which calves are highly prized. Because the tourism industry is also a buyer of elephants, supporting such population growth would also put downward pressure on elephant prices (Fig. 2c).

Overall, our ecological-economic model does not explicitly predict the size of the elephant population in a century. It rather provides a framework for policy-makers, conservationists and managers to anticipate the impacts of socio-economic determinants and their policies —such as exports, or wild elephant conservation— on the fate of the population. Moreover, the interactions between captive and wild pools should be further investigated as they set in motion poorly understood demographic, genetic, epidemiological, economic and socio-cultural processes that strongly influence the species’ population viability.

## Methods

### Study Species and Area

**Figure 4:**
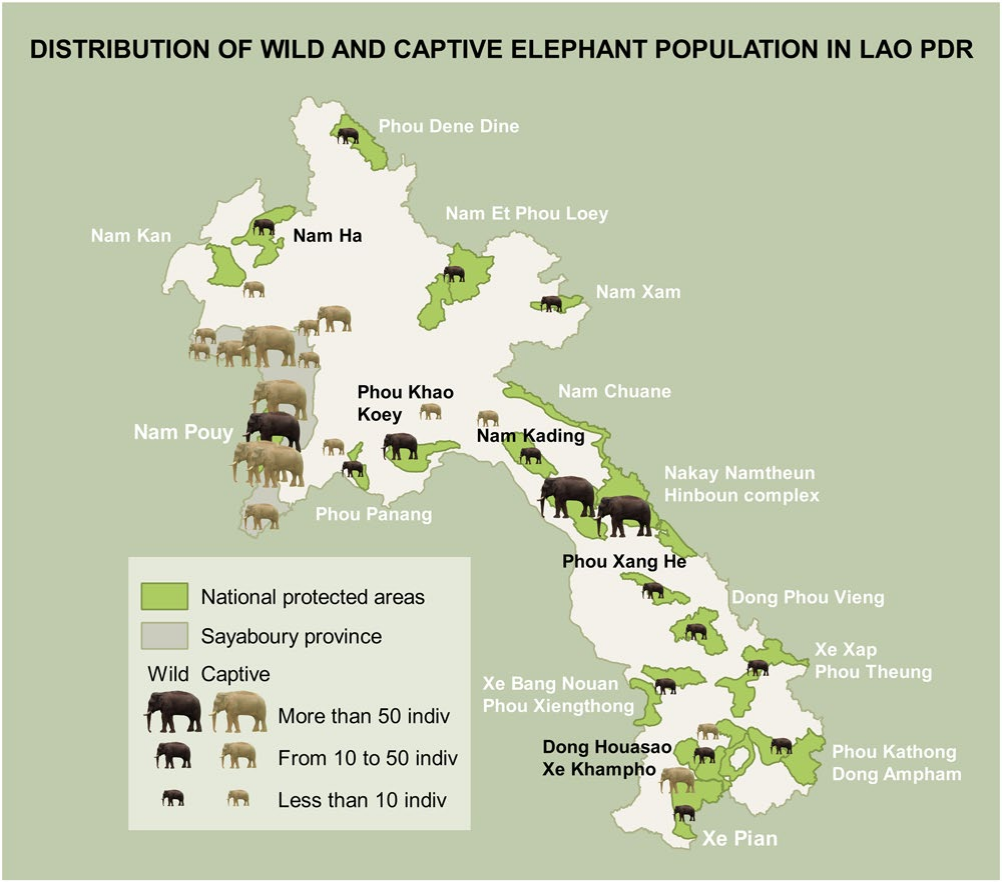
Map of captive and wild elephant populations in Lao PDR. Map has been designed with QGIS version 2.14.0-Essen (www.qgis.org) and Photoshop version 9.0 (www.adobe.com). Protected areas shape files are downloaded from The World Database on Protected Areas (WDPA), IUCN and UNEP-WCMC (2017), available at: www.protectedplanet.net.

Laos has a centuries-long tradition of raising Asian elephants^23^. Most captive elephants live in remote areas and are used to extract logs in the forestry industry^27,33^. After their daily working hours, they are tied to trees where they can forage by themselves and are moved twice a day by their keepers. Keepers also often release their elephants in the forest for a few months while the keepers are engaged in rice farming^34^. Most of the country’s captive elephants live in Sayaboury province (Fig. 4), which also hosts the Nam Pouy National Park (Lat: 18°12′–18°58′N, Long: 101° 04′–101°32′E) – home of the second largest wild population in the country^23,27,34^.

### Population data

Since 2006, the Lao Care and Management Program (LECMP) of the Department of Livestock and Fisheries of Lao PDR has implemented a national campaign to register all captive elephants. Captive elephants are microchipped by the LECMP. Their records, including name, microchip number, sex, age, physical description, medical history, ownership details and location, are saved in a database. Data are updated twice-yearly during LECMP veterinary care visits. We used the data on captive populations structured by sex and age extracted from the database with the year 2012 as a reference^21^. Data on the Nam Pouy wild elephant population from 1990 to 2012 were extracted from the literature^23–25^. More details on the data used throughout are provided in Supplementary Note 4.

### Trade in captive elephants

Captive elephants are privately owned in Laos. Domestic trade is allowed after the payment of a tax to local authorities^26^. Elephant prices are driven by demand on the domestic market. Domestic sales or re-sales do not change the size of the population unlike exports. CITES Convention does not allow international trade in elephants. Despite its ratification by the Lao government in 2004, the export of living elephants continues. Juvenile females illegally cross the border into Thailand and China^9^. National authorities also export young elephants in the form of “rentals” or diplomatic gifts^21^. Being either legal or illegal, exports are considered as permanent, individuals are permanently removed from the captive population.

### Breeding practices in Lao PDR

We studied mahout (elephant handler) practices and their impacts on wild-captive interactions using ethno-ecological methods. The topics covered in this study included the perception of the species and its wild-captive status, the description of husbandry practices, including working, temporary releasing, breeding and interacting with the wild pool, and the respondent perception of the future. In particular, this study described the free-grazing and husbandry practices in Laos that are potentially interfering with the wild elephant pool, and described the mating systems between captive mates. The breeding practices observed during the ethno-ecological study served as a basis to model the drivers of captive population fecundity. Another article is in preparation that will present the global results of the ethno-ecological study.

In 2015, we conducted 52 semi-structured interviews among elephant owners, handlers, and officials throughout the distribution of captive elephants in Laos. Interviews were conducted in Lao language by the first author, recorded on a Zoom H1 handy recorder, and fully translated into French and English. All methods were carried out in accordance with the guidelines for anthropological surveys^35^, and in accordance with the legal requirements of France and the National Center for Scientific Research (CNRS) institutional protocols for research. Research protocol has been approved by Montpellier University. All participants provided informed verbal consent before participation. Sampling was done using a stratified method based on the district of residence and ownership status of the respondent. We coded answers using MaxQDA Analytics Pro 12 software for qualitative data analysis.

### Demographic model for the captive population

**Figure 5:**
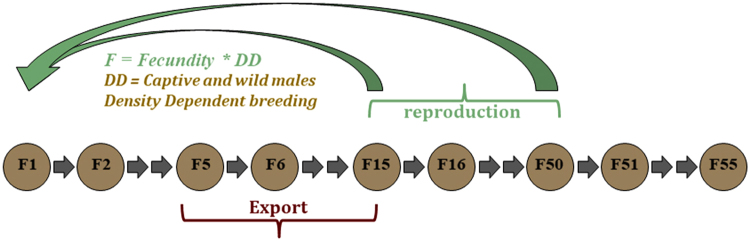
Life cycle of captive females.

We developed a population model composed of a two-sex and 55-age-class matrix to account for the sexual dimorphism in vital rates and sex-specific animal handling practices (Fig. 5).

#### Survival

Demographic data from Laos did not allow estimation of survival rates. We used sex- and age-specific survival rates for a semi-captive elephant population in neighboring Myanmar that shares similar working practices^36^. Survival is given per year from age 0 to 55 with *sf* ^*n*^ the female survival at age n and *sm* ^*n*^ the male survival (see data in Supp. Note 4).

#### Exports

From a population perspective, exports to foreign countries are considered permanent migration. We apply an export function to survival for individuals aged 5 to 15 years with *sf* ^*n*^ *(1-ef)* for females and *sm* ^*n*^ *(1-em)* for males. We defined 2 scenarios: a ban on exports (*ef* and *em* equal 0) and the reference scenario showing the current level of exports. Since females are preferred to males due to their docility and reproductive potential, the respective rates for *ef* and *em* are set to 0.04 and 0.01 for the reference scenario.

#### Birth

Yearly recruitment is given by the product (*sf* ^0^ *sr d F*) for females aged from 15 to 50, with *sf* ^0^ the female survival before their first year, *sr* a balanced sex ratio at birth, *d* the density dependence function and *F* the fecundity function, described hereafter. The same formula is used for males with *sm* ^0^ as the male survival rate.

#### Density-dependent reproduction

**Figure 6:**
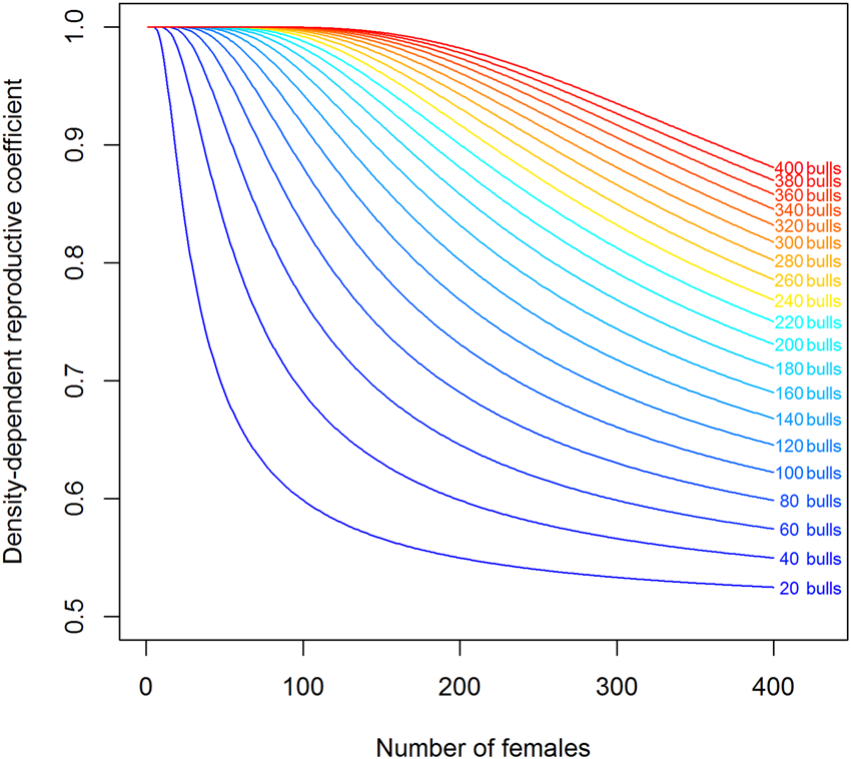
Density-dependent reproductive coefficient depending on the number of females. The density-dependent reproductive function is plotted for several male breeding stock levels: from 20 males (blue line) up to 400 (red line), at 20-male intervals. For a given male stock (one line in the chart), the density-dependent coefficient decreases when the number of females increases, meaning that fecundity is negatively impacted by the excess of females relative to breeding males.

As captive males are engaged in logging activities, many owners of females do not find male mates. Working males are chained to a tree during their resting time and are therefore not available for mating. A prior breeding agreement between female and male owners is therefore required. But female owners found the costs of stud fees too expensive and male owners are reluctant to engage their males in mating because mating is time-consuming and believed to reduce a male’s working strength. Most births therefore result from mating between a captive female and a wild male from Nam Pouy Park. When the breeding process depends on external factors, such as the availability of captive males or the presence of a wild mating pool, the fecundity function is weighted by a coefficient *d* ^37^. We introduced a density-dependent reproductive function in relation to the proportion of the mating pool^38^ where the probability for a captive female to reproduce is dependent on the ratio of the number of breeding males from the captive and wild pools to the number of captive breeding females:

1 $$d=\frac{1}{1+{e}^{-b\frac{Nm}{Nf}}}$$

with *Nf* the number of captive females of breeding age (15 to 50 years), *Nm* the number of captive breeding males from 30 to 55 years plus the estimated number of wild males living in Nam Pouy Park, and *b* the stretching exponent of the function. The parameter *b* allows the growth of the function to be adjusted. In the case of this long-lived polygynous species, we set the stretching exponent to 2. Thus, for a given population of 200 reproductive females and 20, 60 or 100 breeding males respectively, fecundity will be affected by a coefficient *d* equal to 0.57, 0.71 or 0.92, illustrating a steep decrease in the reproductive potential due to the unavailability of males (Fig. 6).

### Population dynamics of the wild pool

The reproduction of captive females depends partially on the Nam Pouy wild pool, as explained above. Given the limited information available on this population, we model the change in Nam Pouy wild population using a population growth rate *wm(t* + *1)* = *grw* × *wm(t)*, with *wm(t)* the total population at time t (*wm(0)* = 68 the population at time 0) and *grw* the yearly average growth rate of Nam Pouy population. We select three different growth rates to explore how wild elephant conservation policies could impact the captive dynamics: an increasing wild pool at a rate of 3% per year, a stable wild pool, and a declining wild pool at a rate of 8% per year. This annual decrease of 8% reflects the sharp decline of this population over the last 20 years (see data in Supplementary Note 4).

### Economic decision-making

According to microeconomic theory, owners tend to maximize profit. Because breeding is not compatible with female elephants working, elephant owners make an economic choice between having their female elephants breed or work. Thus, our economic model assumes owners maximize expected profits by choosing whether or not to breed their females, where the expectation arises because owners do not know future elephant prices or logging income with certainty. This decision framework implies a decision rule where owners will choose to breed up until the expected net present value of breeding exactly equals the opportunity cost of breeding (i.e., foregone timber income). This is equivalent to choosing to breed until the profit ratio (2) is equal to or greater than 1.

At any point in time, the owner compares how much they could receive in net present value by selling a calf after weaning (*Price*_*t+*3_), knowing that the survival at 3 years (*surv*_*3*_) is 0.75, with the alternative income from logging during the 3 years. The profit ratio *P* is therefore:

2 $$P=[sur{v}_{3}\ast \frac{Pric{e}_{t+3}}{{(1+r)}^{4}}]/[\frac{In{c}_{t}}{1+{\rm{r}}}+\frac{In{c}_{t+1}}{{(1+r)}^{2}}+\frac{In{c}_{t+2}}{{(1+r)}^{3}}]$$

with *r* = 10%, the discount rate that takes into account the time value of money or the risk aversion for agricultural projects in Asian developing countries^39–41^. Thus, a profit ratio greater than 1 implies there is an economic incentive to breed females, whereas a profit ratio less than 1 implies that the opportunity cost of foregone logging profits is too large to justify breeding. More information on owners’ price estimation methods are given in Supplementary Note 2. Finally, the costs related to each activity do not appear in the calculation of the profit because they are limited – elephants are foraging freely in Laos, and the cost of guarding is identical with or without a calf. As a result, feeding and labor costs of the two alternatives do not influence the marginal decision to breed or not.

All monetary values, including incomes and profit, have been converted to constant US dollars (USD) in the year 2000 using the methods of Williamson^42^ in order to correct prices for the high inflation in Laos.

#### Price and Income

According to supply and demand theory, the price of an elephant can be expressed in terms of the population size given by the demographic components of the model. From the ethno-ecological study conducted among 26 owners who sold elephants between 1994 and 2012, we assigned each reported price to the population estimate given by the model at the time of sale. Elephant price in constant USD follows an exponential relationship:

3 $$Price={v}_{1}{e}^{{v}_{2}population}$$

with *v*_1_ (Est. = 119200, t = 2.234, p = 0.035) and *v*_2_ (Est. = −0.003633, t = −4.697, p ≪ 0.01).

Working incomes are estimated from IC Suter’s 2011 survey^27^ among 133 elephant owners and keepers, which indicated that average income was equivalent to 4,070 constant USD (i.e., 6.11 times the constant per capita Gross Domestic Product (GDP)). Economists generally use GDP per capita as an indicator of changes in household incomes^43^.

To estimate prices and incomes after 2012, we applied an average economic growth rate (eco_t_) to the owner income and price of an elephant according to the long-term GDP projections from the Asian Development Bank^44^. Moreover, we added stochastic variation to the projected economic growth rate following a normal distribution with mean 1 and a variance of 0.01. Thus, from 2012 and forward, prices at time *t* equal: $Future\,price{s}_{t}=Reg(pric{e}_{t})\prod _{j=1}^{t}ec{o}_{j}\ast {\mathscr{N}}(1,0.01)$, with Reg(Price) the price function (Equation 3). Income at time *t* + *1* is given by $Incom{e}_{t+1}=Incom{e}_{t}\ast ec{o}_{t}\ast {\mathscr{N}}(1,0.01)$.

### Linking fecundity with elephant owners’ profit

**Figure 7:**
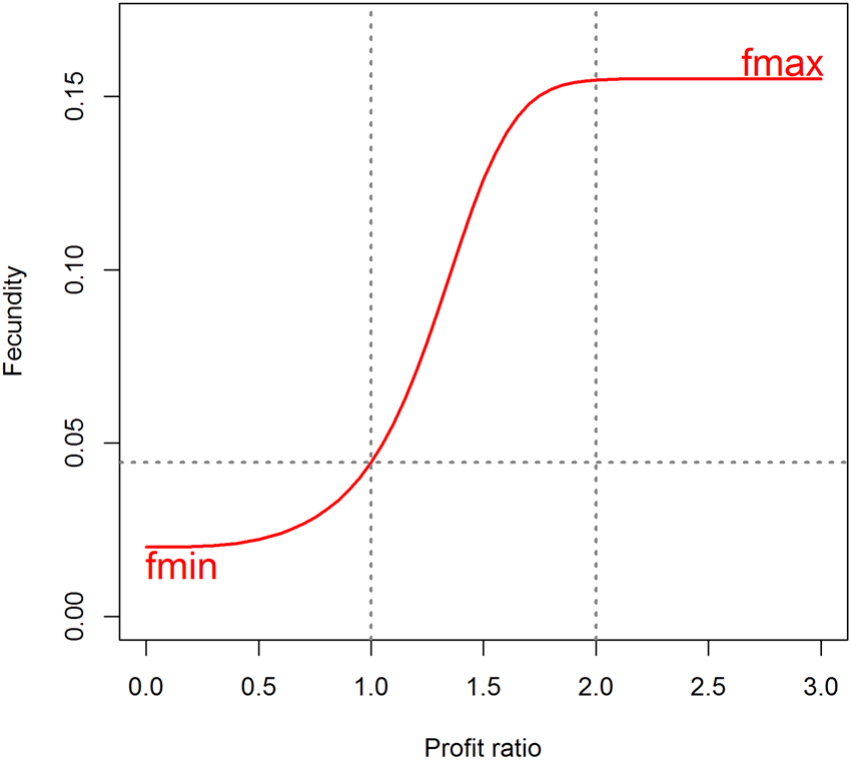
Fecundity as a function of the owner’s profit. Profit is the ratio of the calf’s selling price to the income generated by the foregone work. Fecundity is maximum when breeding generates twice as much as working.

Fecundity is driven by the financial incentive of owners to breed their elephants instead of having them work. When working income exceeds that of breeding (profit ratio *P* < 1), the incentive to breed is low and fecundity is minimal or opportunistic (*f*_*min*_). Fecundity increases as profit increases up to a maximum (*f*_*max*_) set by the biological constraints of the species (Fig. 7). Thus, we model fecundity (F) as a logistic function:

4 $${\rm{F}}={f}_{max}\frac{1}{1+(\frac{{f}_{max}}{{f}_{min}}-1){e}^{-{P}^{\sigma }}}$$

where *P* is the profit ratio (2). The function is bounded respectively by *f*_*max*_ the maximal fecundity and *f*_*min*_ the opportunistic fecundity. The parameter σ is used to fit the steepness of the function^45^ so that fecundity is maximized when the profit ratio equals 2. We retained a profit ratio of 2 for *f*_*max*_ to account for the most extreme case in breeding arrangements (i.e., where all owners consider it profitable to breed their females). Such a rate is a strong incentive for any owner able to pair his female with a wild male. However, the owner of a captive sire usually claims a maximum of one third of the calf value or calf ownership as stud fee, leaving 2/3 of the calf price to the female’s owner (Netvalue = 2/3). In this case the profit for the female’s owner should be positive if: *Price* ∗ *surv*_3_ ∗ *Netvalue* ≥ ∑^3^_t =1_ *Inc*_*t*_ (derived from Equation 2), or profit ratio P ≥ 2. As a result, parameter values used in our model are f_max_ = 0.155^10^, f_min_ = 0.02^21^, and σ = 3.

### Sensitivity and elasticity analyses

The sensitivity analysis allows studying the effect of variation in the model parameters on the population dynamics as measured by their asymptotic growth rate (λ). The asymptotic population growth rate, calculated as the dominant eigenvalue of the matrix, is calculated using the R popbio package^46^. We used elasticities instead of sensitivities because these metrics take into account the unit in which the demographic parameters are measured and thus make comparisons possible between these parameters. The elasticity (EL) of parameter α is given by:

$${\rm{EL}}(\alpha )=\frac{(\lambda ^{\prime} -\lambda )}{\lambda (1+{\rm{\Delta }}{\rm{\alpha }})}$$

where λ′ is the new value of λ after applying a variation Δ to the parameter α.

### Model calibration

**Figure 8:**
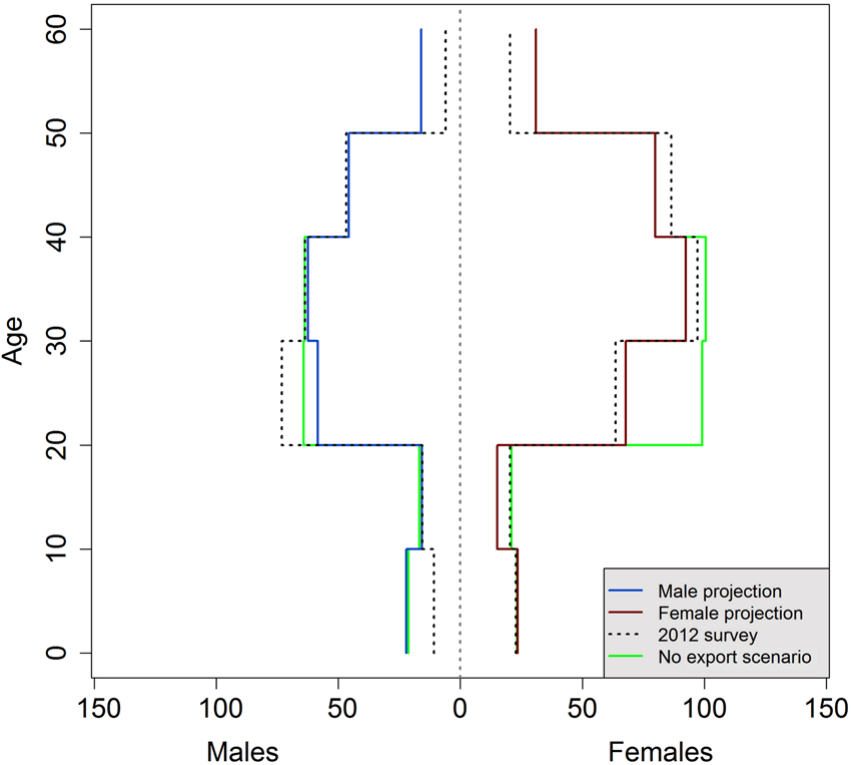
Age pyramid of captive elephants in 2012. Dotted lines show the number of males and females given by the 2012 census. Solid lines indicate the results of the model projection for year 2012. The green line shows the structure of the population in absence of export, confirming the effect of this practice on the number of females aged from 20 to 30 years.

To calibrate our model, we projected the population over 20 years starting from a stable structured population of one thousand individuals in 1992 and compared its demographic structure with the observed structure in 2012. Population abundance reached 530 individuals, consistent with 2012 estimate of 527 individuals. Moreover, the population structure is highly transient and unstable. A strong bias is observed in favor of elephants over 20 years old and a deficit of females in the 20–29 years age class resulting from our hypotheses (sharp drop in fecundity and export of juvenile females during the last twenty years). The population structure derived in the model (Fig. 8) cannot be distinguished from the structure inferred from the 2012 census data (Chisq = 13.6, df = 11, p = 0.25). All analyses were performed with R version 3.2.3^47^.

### Data Availability

All data generated or analysed during this study are included in this published article (and its Supplementary Information files).

## Electronic supplementary material

Supplementary notes
