# Supplementary material for: Wild-captive interactions and economics drive dynamics of Asian elephants in Laos
Source: Sci Rep. 2017 Nov 1;7:14800. doi: 10.1038/s41598-017-13907-x (PMC5665948; doi:10.1038/s41598-017-13907-x)
Supplement: Supplementary file 1 — Supplementary notes [file 41598_2017_13907_MOESM1_ESM.pdf]

Supplementary Information

Wild-captive interactions and economy drive Asian elephant dynamics in Laos

Authors: Gilles Maurer[1,2,*], Benjamin Rashford[3], Vatsana Chanthavong[4,5], Baptiste Mulot[2], Olivier Gimenez[1].

[1] Centre d'Ecologie Fonctionnelle et Evolutive (UMR 5175), CNRS – Université de Montpellier – Université Paul-Valéry Montpellier – EPHE, campus CNRS, Montpellier, France
[2]Zooparc de Beauval & Beauval Nature, Saint-Aignan, France
[3]Department of Agricultural and Applied Economics, University of Wyoming, Laramie, USA
[4] Department of Livestock and Fisheries, Ministry of Agriculture and Forestry, Vientiane, Lao PDR
[5] ElefantAsia non-profit organization, Paris, France
* Corresponding author: gilles.maurer@gmail.com

Supplementary note 1: Breeding practices in Lao PDR

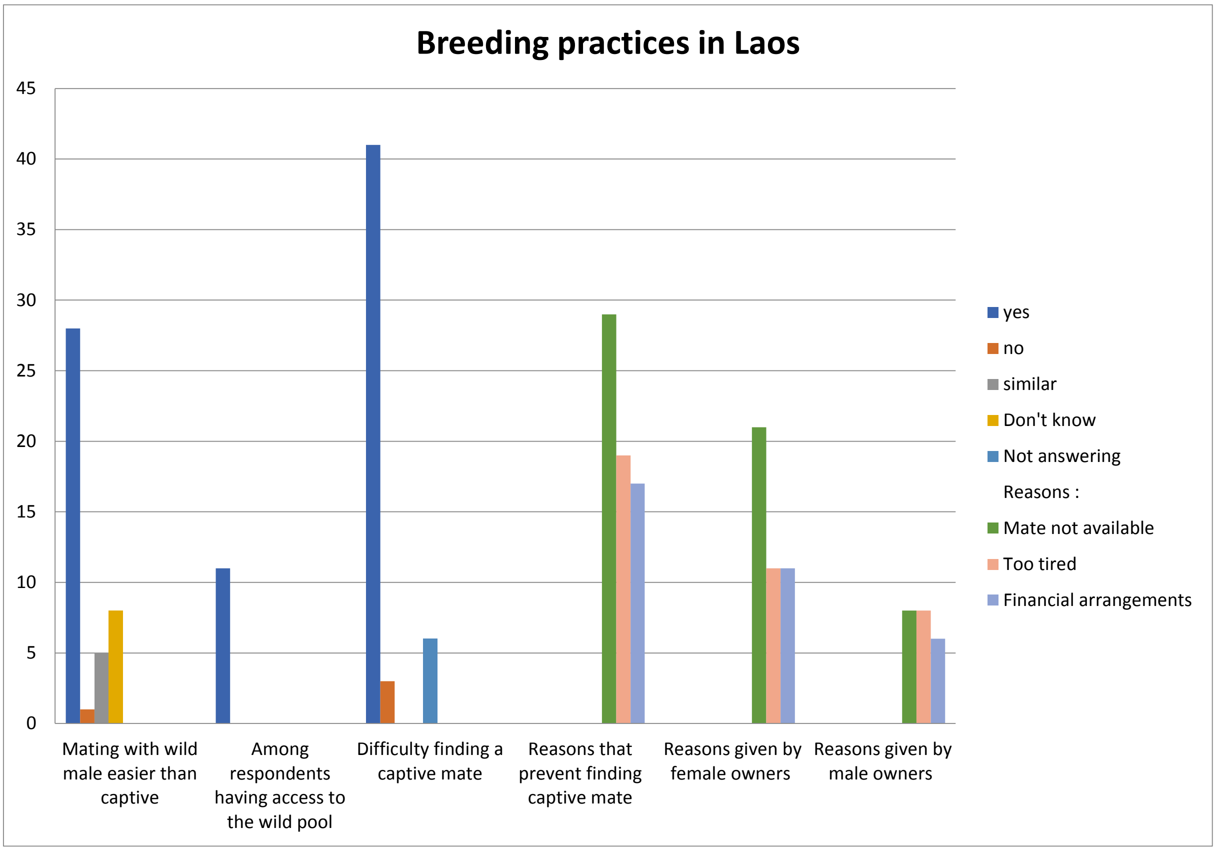

Figure S1: Results from an anthropological study among elephant owners and keepers in Laos

Extracts from semi-structured interviews undertook in 2015 about elephant owners and keepers handling practices and their impact on the wild-captive interactions.

Supplementary note 2: Modelling the owner decision-making process

To obtain projections from our bio-economic model, we developed a model to capture the owner decision-making process. Consistent with economic theory, we assume elephant owners maximize expected utility, where expected utility is wholly determined by expected profits (i.e., profits are the only argument in the owners' utility function, which is a standard in agricultural decision models) and where the only uncertainty stems from future prices and logging income. We also assume that owners make discrete time decisions in each period, but consider the future income stream in making each periods decision. So, an owner decides whether to breed or not by comparing the expected net-present value of profit from breeding to the expected net-present value of profit from not breeding. Considering these two states of the world for each individual female elephant in any decision period, the owner compares:

(1) $E[NPV\ of\ profit\ from\ breeding] = surv_3 * \frac{E[Price_{t+3}]}{(1+r)^4} + \sum_{t=4}^{T} \frac{E[Income_t]}{(1+r)^t}$

with

(2) $E[NPV\ of\ profit\ from\ not\ breeding] = \sum_{t=1}^{T} \frac{E[Income_t]}{(1+r)^t}$.

This expected utility(profit) maximization problem can be explicitly expressed as:

$$Max\ E[\pi] = \delta\left(surv_3 * \frac{E[Price_{t+3}]}{(1+r)^4} + \sum_{t=4}^{T} \frac{E[Income_t]}{(1+r)^t}\right) + (1-\delta)\sum_{t=1}^{T} \frac{E[Income_t]}{(1+r)^t},$$

where $\delta = 1$ if the owner chooses to breed and zero if not. Solving this optimization problem results in an interior equilibrium where: $surv_3 * \frac{E[Price_{t+3}]}{(1+r)^4} = \sum_{t=1}^{3} \frac{E[Income_t]}{(1+r)^t}$, or where the expected net present value of breeding exactly equals the opportunity cost of breeding (i.e., foregone timber income). Thus, owners will optimally choose to breed their elephants (or not) until this condition is met, which is equivalent to choosing to breed (or not) until the profit ratio is equal to 1.

Equation (2) gives the profit ratio *P* of the elephant price over the net-present value of logging income adjusted by the survival at 3 years:

$$P = surv_3 * \left[\frac{Price_{t+3}}{(1+r)^4}\right] / \left[\frac{Inc_t}{1+\mathrm{r}} + \frac{Inc_{t+1}}{(1+r)^2} + \frac{Inc_{t+2}}{(1+r)^3}\right] \quad (2)$$

However, when the owner should make a decision, he does not know the future price of the elephant or his income at time *t* + 3; the different situations that may arise are summarized in the table below:

Time (in years)	t-1 **DECISION**	t	t+1	t+2	t+3 **PROFIT**
Reproduction	mating & 1st year gestation	2nd year gestation	Birth, 1st year nursing	2nd year nursing	3rd year nursing, taming
Income from breeding	Working Income (t-1)	0	0	0	Working Income (t+3) + Calf Price (t+3)
Income from working	Working Income (t-1)	Working Income (t)	Working Income (t+1)	Working Income (t+2)	Working Income (t+3)
Matrix parameters	Elephant price (t-1) & Income (t-1)	Fecundity (t), Price(t), Income(t)	Population Pop (t+1)	-	-

Table S1: Captive breeding timeframe

The mahout relies on the information at his disposal to motivate his choice, i.e. on prices in previous years, using the same approach described for European livestock farmers[1]. Once his decision is made, he depends on the market price at time t + 3. Therefore, to estimate future prices, the mahout applies the average growth rate of the last two years over the coming years:

(i) the annual price increase of an elephant: $$\Delta \mathrm{P} = \left[\frac{Price_{t-2}}{Price_{t-3}} + \frac{Price_{t-1}}{Price_{t-2}}\right]/2 \qquad \text{(S1)}$$

(ii) the annual income increase: $$\Delta \mathrm{I} = \left[\frac{\mathrm{Income}_{t-2}}{\mathrm{Income}_{t-3}} + \frac{\mathrm{Income}_{t-1}}{\mathrm{Income}_{t-2}}\right]/2 \qquad \text{(S2)}$$

From equations (S1) and (S2), the mahout can estimate the future prices and incomes as follows:

$\mathrm{Price}_{t+3} = \Delta \mathrm{P}^4 * \mathrm{Price}_{t-1}$; $\mathrm{Income}_{t} = \Delta \mathrm{I} * \mathrm{Income}_{t-1}$; $\mathrm{Income}_{t+1} = \Delta \mathrm{I}^2 * \mathrm{Income}_{t-1}$ and so on.

We obtain a revised version of the profit ratio (Eqn 2) based on price and income at time *t* - 1 when the mahout makes his decision:

$$profit = surv_3 * \left[\frac{\Delta \mathrm{P}^4 * Price_{t-1}}{(1+r)^4}\right] - \left[\frac{\Delta \mathrm{I} * Income_{t-1}}{1+r}\right] * \left[1 + \frac{\Delta \mathrm{I}}{1+\mathrm{r}} + \left(\frac{\Delta \mathrm{I}}{1+\mathrm{r}}\right)^2\right] \qquad \text{(S3)}$$

List of references

1. Bonnays, G. & Latouche, D. Prix agricoles : baisse sur le long terme mais de fortes fluctuations. *Econ. Stat.* **226,** 27–33 (1989).

Supplementary Note 3: Elasticity analysis

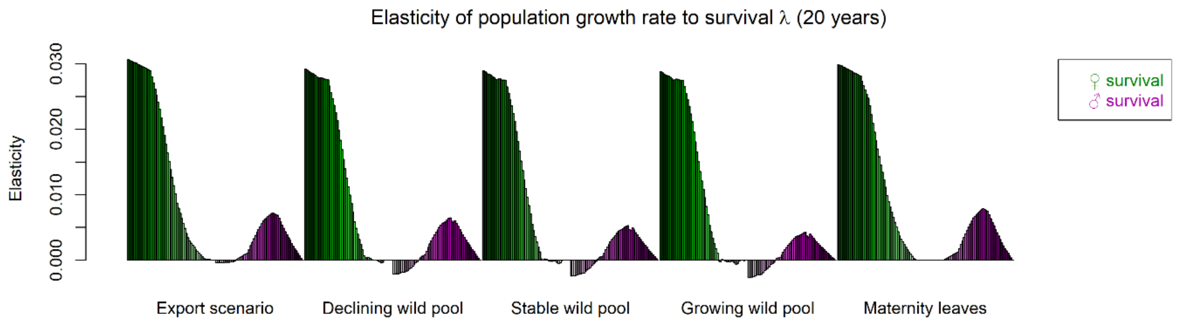

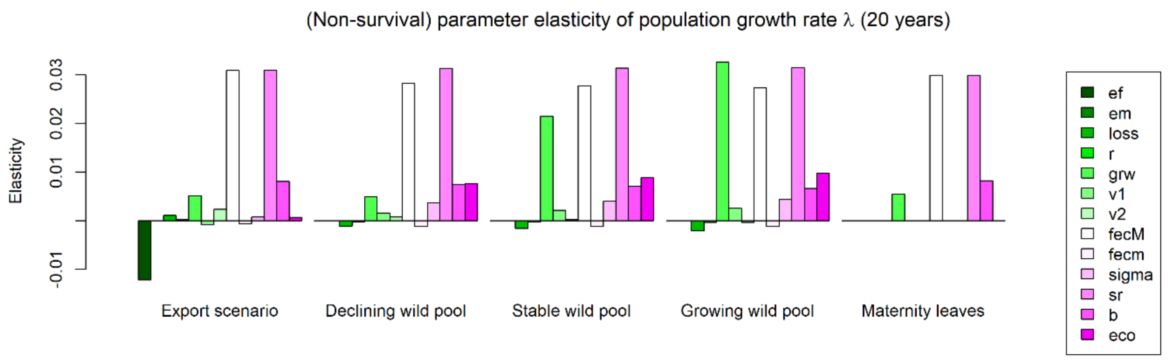

Figure S2: Elasticity of population growth rate (λ) to model parameters (20-year projection)

Elasticity is given for survival from year 1 to 55 (upper chart)
Parameters description (non survival parameters)

ef	Export rate – females
em	Export rate – males
loss	Maternity leave (Income=0)
r	Discount rate
grw	Wild pool growth
v1	Price regression intersect
v2	Price regression slope
fecM	Maximum fecundity
fecm	Minimum fecundity
sigma	Steepness of the fecundity function
sr	Sex ratio
b	Stretching exponent (Density Dep. reprod. Function).
eco	Economic growth rate projections

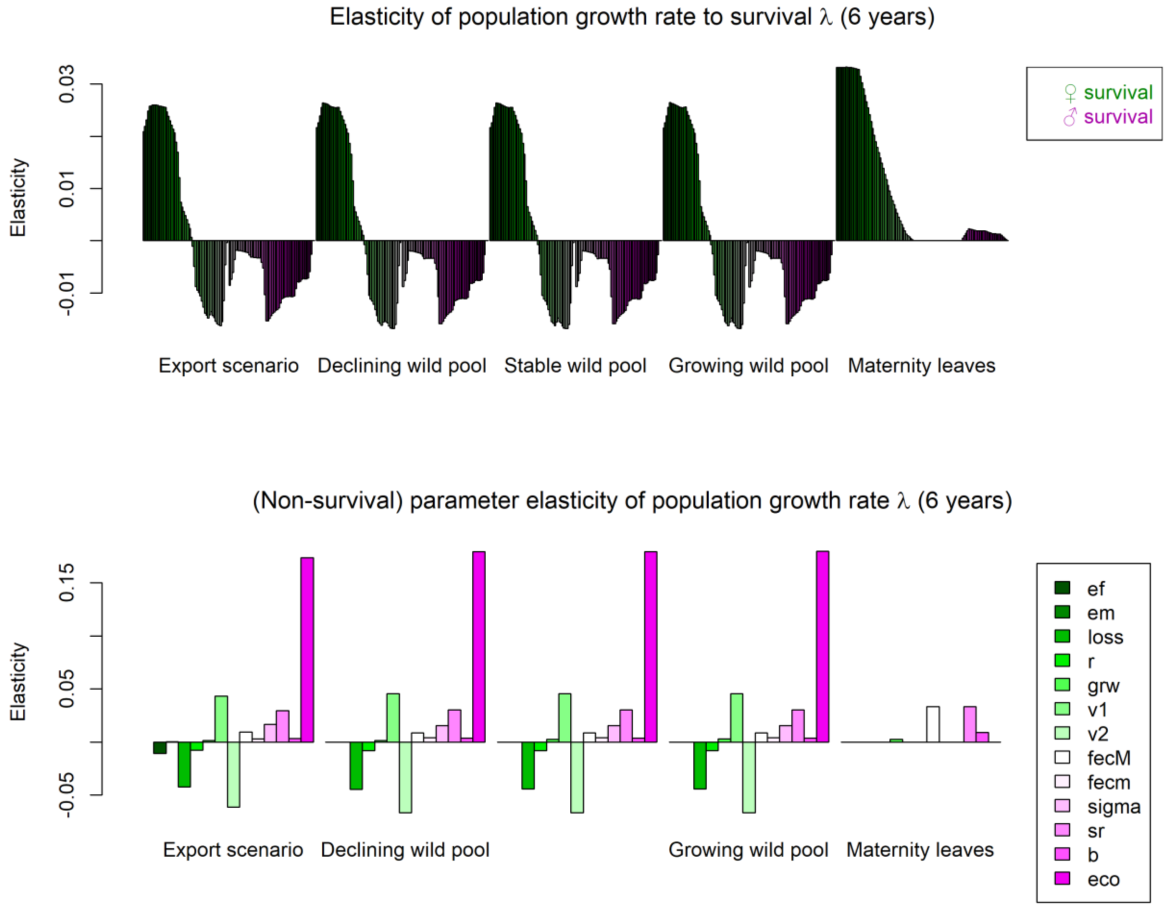

Figure S3: Elasticity of population growth rate (λ) to model parameters (6-year projection)

On the short term (6 years) the population growth is highly sensitive to variation in economic growth (eco) with an elasticity of 0.18 and other economic-related parameters (v1 and v2 prices, loss of income during gestation) showing elasticities around 0.05. On a mid-term basis (20 years), the highest values of elasticities are for fecundity parameters [maximum fecundity (fecM) and sex ratio (sr) – 0.03], the wild pool growing rate [0.033 (growing wild pool) – 0.021 (stable wild pool) – 0.05 (declining wild pool)] and female export rate (0.05). Comparing the relative contribution of the model parameters on two different time scales confirms that socio-economic parameters are strongly impacting the population dynamics on the short term, while demographic rates (export, wild pool, fecundity, survival) are driving the dynamics on the long term.

Supplementary Note 4: Data and model parameters

Selected scenarios

	Female export (ef)	Male export (em)	Wild pool growth (grw)	Maternity leave (Income=0)
i) Reference scenario	0.04	0.01	0.92	1
No export scenarios below:				
ii) Declining wild pool	0	0	0.92	1
iii) Stable wild pool	0	0	1	1
iv) Growing wild pool	0	0	1.03	1
v) Maternity leave	0	0	0.92	0

Table S1: parameters for the selected scenarios

Note 1: Calculation and sources for the Reference scenario (i) are given below.

Note 2: Other scenarios have been tested but not reported here. All scenarios with exports at the given reference rate lead to extinction, whatever the dynamics of the wild pool or maternity-leaves scheme.

Other parameters

	Description	Value	Source/comments
fecM	Maximum fecundity	0.155	From Sukumar, 1997 [2]
fecm	Minimum fecundity	0.02	From Suter, 2011 [3]
sigma	Steepness of the fecundity function	3	fecundity is maximum when the profit ratio equals 2, i.e. stud fees equal income
sr	Sex ratio	0.5	
b	Stretching exponent (Density dependent reprod. Function).	2	From DD in manuscript
wm0	Wild pool at time 0	68	See wild pool estimate below
r	Discount rate	0.1	From Fisher, 2011; Markandya 1991; Tomish, 2001 [4–6]
v1	Price regression intersect	119200	See prices below
v2	Price regression slope	-0.003633	
Inc	Income at time 0	4213	In constant USD from Suter, 2013 [3]
eco	Economic growth rate projections	1.051 (2012)<br>1.04 (+10 years)<br>1.035 (+10 years)<br>1.03 (+10 years)<br>1.025 (+10 years)<br>1,020	From ADB GDP long term projections [7]
Ɲ	Stochastic economic var.	Eco * Ɲ (1,0.01)	mean 1; variance 0.01

Table S2: Other parameters used in the model

Survival rates

Year	Female	Male	Year	Female	Male	Year	Female	Male
0	0.918	0.908	19	0.991	0.991	38	0.973	0.958
1	0.951	0.925	20	0.996	0.981	39	0.959	0.957
2	0.976	0.964	21	0.996	0.99	40	1	0.985
3	0.984	0.975	22	0.988	0.98	41	0.978	0.954
4	0.952	0.918	23	0.992	0.99	42	1	0.968
5	0.946	0.945	24	0.991	0.99	43	0.98	0.983
6	0.976	0.956	25	0.98	0.98	44	0.931	0.966
7	0.99	0.962	26	0.986	0.99	45	0.986	0.947
8	0.992	0.984	27	0.995	0.979	46	0.926	0.981
9	0.995	0.984	28	0.989	0.989	47	1	0.962
10	0.994	0.967	29	0.989	0.978	48	0.972	0.941
11	0.999	0.992	30	0.985	0.978	49	0.962	0.958
12	0.994	0.991	31	0.985	0.977	50	0.962	0.891
13	0.992	0.983	32	0.993	0.977	51	0.962	0.985
14	0.996	0.982	33	0.993	0.976	52	0.962	0.99
15	0.987	0.991	34	0.992	0.976	53	0.962	0.95
16	0.997	0.991	35	0.97	0.963	54	0.962	0.895
17	0.99	0.982	36	0.981	0.974	55	0	0
18	0.996	0.981	37	0.98	0.96			

Table S3 : Survival rates by sex and age
Source : From Mar, 2007 [8]

Female component of the matrix

$$\begin{bmatrix}
0 & 0 & \cdots & \cdots & 0 & sf^0.sr.d.F & sf^0.sr.d.F & \cdots & sf^0.sr.d.F & 0 & \cdots & 0 & 0 \\
sf^1 & 0 & & & & & & & & & & & 0 \\
0 & sf^2 & \cdots & & & & & \cdots & & & \cdots & & 0 \\
\vdots & \ddots & & & & & & & & & & & \vdots \\
 & & sf^6 * (1-ef) & & & & & & & & & & \\
 & & & \ddots & sf^{14} * (1-ef) & & & & & & & & \\
\vdots & & & & & sf^{15} * (1-ef) & & & & & & & \vdots \\
 & & & & & & sf^{16} & & & & & & \\
\vdots & & & & & & & \ddots & sf^{50} & & & & \\
0 & 0 & & & & & & & & sf^{51} & & & 0 \\
0 & 0 & & & \cdots & & & \cdots & & & \ddots & sf^{54} & sf^{55}
\end{bmatrix}$$

Eq. S4: Matrix – female component

The diagram above represents the first 55 columns corresponding to the 55 age classes of females only. The survival of females (sf^n) is expressed by age from 0 to 55 years. Fecundity (F) is applied to the age classes of females between 15 and 50 years with a sex ratio (sr) equal to 0.5 and corrected for survival before the first birthday (sf^0). Female export rate (*ef*) is applied between 5 to 15 years old. The matrix is supplemented by its equivalent for males. Export rate for males is applied to the same age classes (5-15 years) with a rate (*em*).

Data at origin (year 2012)

Captive population in 2012 by sex and age-class

Age class	Female	Male
[1:10]	23	11
[11:20]	20	16
[21:30]	64	73
[31:40]	97	64
[41:50]	86	47
[51:55]	20	7
Total	311	217

Table S4: Captive population numbers by sex and age-class in 2012

Sources : Lao Elephant Care and Management Program (Lao Department of Livestock and Fisheries), data published in Suter, 2014 [9]

Wild pool growth rate estimate for the reference scenario

We modeled the dynamics of the wild pool using geometric growth calculated from published data.

The population growth from time t to time t' equals: $\left(\frac{N_{t'}}{N_t}\right)^{\frac{1}{t'-t}}$.

**Year**	**Mean**	**range**	**Source**
1990	450	400-500	From Phanthavong, 1992 [10]
2000	350		From Duckworth, 1999 [11]
2009	100	100	From Khounboline, 2011 [12]
2011	70	60-80	From Maurer, 2011 [13]

Table S5: Estimates of the Nam Pouy wild population across time

We calculated the average geometric growth (*grw*) over the period 1990-2012 and modeled the population such as $wm(t+1) = grw \times wm(t)$, with $wm(t)$ the total population at time t, $wm(0) = 68$ the population at time 0, and $grw = 0.92$ the average growth rate of the population. This annual decrease of 8% illustrates the sharp decline of this population over the last 20 years.

Elephant prices

Year	1994	1997	1999	2006	2007	2008	2009	2010	2011	2012	2013
USD	7830	6690	5010	9330	9960	18300	15800	11500	15400	17000	19000
			9740		8900			9420	16800	17900	
					12200			13700		19300	
					8770			13300		25000	
								14100			
								14300			
								14900			
								11900			

Table S6: Elephant prices in constant USD

Source : Ethno-ecological study, prices have been converted in constant USD in the year 2000 using the methodology of Williamson [14].

List of references

1. Bonnays, G. & Latouche, D. Prix agricoles : baisse sur le long terme mais de fortes fluctuations. *Econ. Stat.* **226,** 27–33 (1989).

2. Sukumar, R., Krishnamurthy, V., Wemmer, C. & Rodden, M. Demography of Captive Asian Elephants (Elephas maximus) in Southern India. *Zoo Biol.* **272,** 263–272 (1997).

3. Suter, I. C., Hockings, M. & Baxter, G. S. Changes in Elephant Ownership and Employment in the Lao PDR: Implications for the Elephant-Based Logging and Tourism Industries. *Hum. Dimens. Wildl.* **18,** 279–291 (2013).

4. Fisher, B., Edwards, D. P., Giam, X. & Wilcove, D. S. The high costs of conserving Southeast Asia's lowland rainforests. *Front. Ecol. Environ.* **9,** 329–334 (2011).

5. Tomish, T., Noordwijk, M. van & Budidarsono, S. in *Tradeoffs or synergies ?* (eds. Lee, D. . & Barret, C. B.) 221–245 (Centre for Agricultural Bioscience International, 2001).

6. Markandya, A. & Pearce, D. W. Development, the Environment, and the Social Rate of Discount. *World Bank Res. Obs.* **6,** 137–152 (1991).

7. Asian Development Bank. *Long term projections of Asian GDP and trade*. (2011).

8. Mar, K. U. The demography and life history strategies of timber elephants in Myanmar. (University College London, 2007).

9. Suter, I. C., Maurer, G. & Baxter, G. Population viability of captive Asian elephants in the Lao PDR. *Endanger. Species Res.* **24,** 1–7 (2014).

10. Phanthavong, B. & Santiapillai, C. Conservation of elephants in Laos. *Asian Elephant Spec. Gr. Newsl.* **8,** 25–48 (1992).

11. Duckworth, J. ., Salter, R. . & Kounboline, K. *Wildlife in Lao PDR: 1999 status report*. IUCN, Lao PDR, Vientiane (1999).

12. Khounboline, K. Current Status of Asian Elephants in Lao PDR. *Gajah* **35,** 62–66 (2011).

13. Maurer, G. & Bouchard, B. *Current situation of elephants in Lao PDR.* International conference on elephant and wildlife health management in Asia. (Kasetsart university, Bangkok, 2011).

14. Williamson, S. H. & Officer, L. H. Seven Ways to Compute the Relative Value of a U.S. Dollar Amount, 1774 to present. *MeasuringWorth* (2010). at www.measuringworth.com/worthmeasures.php
